# Log odds of positive lymph nodes is prognostically equivalent to lymph node ratio in non-metastatic colon cancer

**DOI:** 10.1186/s12885-020-07260-y

**Published:** 2020-08-14

**Authors:** Ali Riaz Baqar, Simon Wilkins, Wei Wang, Karen Oliva, Paul McMurrick

**Affiliations:** 1 Department of Surgery, Cabrini Hospital, Cabrini Monash University, Malvern, VIC 3144 Australia; 2 Department of Epidemiology and Preventive Medicine, Monash University, Melbourne, VIC 3004 Australia; 3 Cabrini Institute, Cabrini Hospital, Malvern, VIC 3144 Australia

**Keywords:** Colon cancer, Lymph nodes, Patient outcomes

## Abstract

**Background:**

Globally, colorectal cancer (CRC) is the third and second leading cancer in men and women respectively with 600,000 deaths per year. Traditionally, clinicians have relied solely on nodal disease involvement, and measurements such as lymph node ratio (LNR; the ratio of metastatic/positive lymph nodes to total number of lymph nodes examined), when determining patient prognosis in CRC. The log odds of positive lymph nodes (LODDS) is a logistic transformation formula that uses pathologic lymph node data to stratify survival differences among patients within a single stage of disease. This formula allows clinicians to identify whether patients with clinically aggressive tumours fall into higher-risk groups regardless of nodal positivity and can potentially guide adjuvant treatment modalities. The aim of this study was to investigate whether LODDS in colon cancer provides better prognostication compared to LNR.

**Methods:**

A retrospective study of patients on the prospectively maintained Cabrini Monash University Department of Surgery colorectal neoplasia database, incorporating data from hospitals in Melbourne Australia, identified patients entered between January 2010 and March 2016. Association of LODDS and LNR with clinical variables were analysed. Disease-free (DFS) and overall (OS) survival were investigated with Cox regression and Kaplan–Meier survival analyses.

**Results:**

There were 862 treatment episodes identified in the database (402 male, 47%). The median patient age was 73 (range 22–100 years). There were 799 colonic cancers and 63 rectosigmoid cancers. The lymph node yield (LNY) was suboptimal (< 12) in 168 patients (19.5%) (*p* = 0.05). The 5-year OS for the different LNR groups were 86, 91 and 61% (*p* < 0.001) for LNR_0_ (655 episodes), LNR_1_ (128 episodes) and LNR_2_ (78 episodes), respectively. For LODDS, they were 85, 91 and 61% (*p* < 0.001) in LODDS_0_ (569 episodes), LODDS_1_ (217 episodes) and LODDS_2_ (75 episodes) groups (*p* < 0.001). Overall survival rates were comparable between the LNR and LODDS group and for LNY < 12 and stage III patients when each were sub-grouped by LODDS and LNR.

**Conclusion:**

This study has shown for that the prognostic impact of LODDS is comparable to LNR for colon cancer patients. Accordingly, LNR is recommended for prognostication given its ease of calculation.

## Background

Globally, colorectal cancer (CRC) is the third and second leading cancer in men and women respectively with 600,000 deaths per year [1]. Nodal status in surgical oncology can be used to assist in prognostication [2], guide decision making regarding adjuvant chemotherapy [3] and the number of examined lymph nodes examined or the lymph node yield (LNY) can be used as a marker for the quality of an oncological resection [4]. In CRC surgery, harvesting a minimum of 12 lymph nodes has been set as an acceptable benchmark. If the LNY is below 12, this has been suggested to be correlated with under-staging of the disease [5].

Lymph node ratio (LNR) (defined as the ratio of metastatic lymph nodes to the total number of lymph nodes examined) has been investigated as an adjunct parameter to conventional nodal staging. The LNR aids in prognosis and for identifying high-risk patients [6]. However, in node-negative colon cancer, which accounts for approximately 75% of patients who have surgery for colon cancer, LNR is zero and is the same as the pN0 classification and therefore does not provide any additional prognostic information [7].

Traditionally, clinicians have relied solely on nodal disease involvement (including the total number of positive lymph nodes) when determining patient prognosis in CRC [8]. Biologically aggressive tumours however, can initially be placed in the same stage as less clinically aggressive tumours, irrespective of nodal disease. The log odds of positive lymph nodes (LODDS) is a logistic transformation formula that uses pathologic lymph node data to stratify survival differences among patients within a single stage of disease. This formula allows clinicians to identify whether patients with clinically aggressive tumours fall into higher-risk groups regardless of nodal positivity and can potentially guide adjuvant treatment modalities.

Recently LODDS has been proposed as a novel prognostic index in colonic and non-colonic cancers [9–11]. In all of these studies, the classification of lymph node status by LODDS proved to be a powerful prognostic indicator with a strong ability to identify patients with a homogeneous prognosis, regardless of lymph node status and count. The aim of this study was to investigate the prognostic impact of LODDS and compare the survival of patients classified in LNR and LODDS groups who underwent a colonic cancer resection.

## Methods

The prospectively maintained Cabrini Monash University colorectal neoplasia database [12] which contains a representative case mix of patients from both the public and private health sector, was examined for consecutive patients treated for colon adenocarcinoma under the care of 11 colorectal surgeons at Cabrini and Alfred hospitals (Melbourne, Victoria, Australia) between January 2010 and March 2016. Data extracted from the database included patient demographics, tumour characteristics, lymph node yield, medical co-morbidities, and oncological end points (local and distal recurrence, overall survival). Patients were divided into groups according to their LNR and LODDS. Survival analysis was performed and compared for the subgroups within LODDS and LNR. Patients who presented with synchronous colonic tumours, metastatic disease, and ASA 5 (American Society of Anesthesiologists) were excluded.

The LNR was defined as the number of positive lymph nodes divided by the total number of lymph nodes harvested. Patients were divided into three LNR groups based on previous literature [9]: LNR_0_ (< 0.05), LNR_1_ (0.05–0.20) and LNR_2_ (> 0.20). At least 12 harvested lymph nodes were accepted as an adequate number and tumour staging was performed according to the seventh edition of the AJCC TNM manual [13]. Pathological examination of lymph nodes in resected specimens relied on manual dissection by the pathologists. A low LNY was defined as fewer than 12 lymph nodes in the resected specimen.

LODDS is defined as the log of the ratio between the number of positive lymph nodes and the number of negative lymph nodes. LODDS is calculated using an empirical logistic transform formula: log (positive nodes + 0.5)/(total nodal count - positive nodes + 0.5). Patients were divided into three groups based on published LODDS studies specific to colorectal neoplasia [9]: LODDS_0_ (<− 1.36), LODDS_1_ (− 1.36 to − 0.53) and LODDS_2_ (> − 0.53).

Surveillance after surgery involved clinical examination, computed tomography (CT) scan of the chest, abdomen and pelvis, colonoscopic visualisation of the residual colon and carcinoembryonic antigen (CEA) levels, all performed at varying intervals post-surgery. Radiology and/or histological studies were used to diagnose local recurrence or distant metastasis. The follow-up was conducted until July 2016. The primary outcomes for the study were overall survival and disease-free survival.

### Statistical analysis

Data analysis was performed using the R 3.5.1 (Windows) statistical package [14]. The effects of clinical variables, LODDS and LNY on disease-free survival (DFS) and overall survival (OS) were investigated using survival analysis techniques such as Kaplan-Meier and log-rank tests. Independent prognostic factors were identified in both univariate and multivariate analyses (Cox regression). The significance level was set at 5%, and terms were included in the models when the *p* value was below this level. *P* < 0.05 was considered statistically significant.

Power calculation was carried out based using the R statistical package [14]. The covariates of interest used in the estimation were LNR and LODDS groups together with additional predictors. With the number of episodes of 862, a significance level of 0.05, and a number of different sets of parameters for overall survival and disease-free survival for LNR and LODDS models, the estimated powers were 91.69% for LNR model and 93.39% for LODDS model for the overall survival, while the powers of disease-free survival were 92.29% for the LNR model and 93.04% for the LODDS model. Therefore, there was sufficient power for the study.

## Results

Between January 2010 and March 2016, a total of 862 treatment episodes were identified from 856 patients on the colorectal neoplasia database. Patient demographics identified 402 men (47%) and the median age of the cohort was 73 (range 22–100) years. The highest percentage of cancer localization occurred in the sigmoid colon (25.2%) and the ascending colon (20.0%). The LNY was ≥12 in 694 episodes (80.5%). The median duration of follow-up was 27.1 (range 0.1–71) months. Patient characteristics and clinicopathological features are summarised in Table 1.

**Table 1:** Patient and tumour characteristics and 5-year overall survival

Variable		N (%)	5-year OS (95% CI)	***p***-value
**N (1st episode)**		***n*** **= 856**		
**Sex**	Male	402 (47.0)	82 (73.5, 91.5)	0.4
	Female	454 (53.0)	86 (80.2, 92.2)	
**Age at first surgery**	< 60	146 (17.1)	91.5 (82.9, 100)	**< 0.001**
	60–79	424 (49.5)	91.3 (87.1, 95.7)	
	≥ 80	286 (33.4)	65.4 (51.1, 83.7)	
**N (All episodes)**		***n*** **= 862**		
**Tumour site**	Caecum	143 (16.6)	77.4 (67.4, 89)	< 0.001
	Ascending colon	172 (20.0)	69 (51.4, 92.6)	
	Hepatic flexure	61 (7.1)	92.4 (82.8, 100)	
	Transverse colon	130 (15.1)	83 (72.9, 94.6)	
	Splenic flexure	43 (5.0)	n/a	
	Descending colon	33 (3.8)	n/a	
	Sigmoid colon	217 (25.2)	98.9 (97.3, 100)	
	Rectosigmoid	63 (7.3)	82.8 (68.6, 100)	
**T stage**	0–2	327 (37.9)	93.3 (88.9, 97.9)	**< 0.001**
	3	451 (52.3)	80.4 (71.5, 90.4)	
	4	84 (9.7)	n/a	
**N stage**	0	619 (71.8)	85.2 (79, 91.9)	**0.01**
	1	165 (19.1)	85.3 (74.2, 98.1)	
	2	78 (9.0)	71.2 (57.4, 88.4)	
**ASA**	1	151 (17.5)	95.9 (90.0, 100)	**< 0.001**
	2	348 (40.4)	91.0 (86.6, 95.7)	
	3	315 (36.5)	73.7 (60.2, 90.3)	
	4	47 (5.5)	46.1 (28.9, 73.6)	
**Pathological grade**	Undifferentiated	5 (0.6)	n/a	**0.009**
	Poor differentiation	164 (19.0)	74.7 (64.5, 86.6)	
	Moderate differentiation	579 (67.2)	85.3 (78.1, 93)	
	Well differentiated	47 (5.5)	91.7 (77.3, 100)	
**LVI**	No	591 (68.6)	84.9 (78.5, 91.8)	**0.02**
	Yes	244 (28.3)	81.7 (74.6, 89.4)	
**CRM**	Negative > 1 mm	465 (53.9)	79.6 (69.3, 91.4)	0.4
	Positive ≤1 mm	8 (0.9)	n/a	
	Not reported	375 (43.5)	86.6 (81.8, 91.6)	
**LNY**	<12	168 (19.5)	85.4 (78.6, 92.9)	0.5
	≥12	694 (80.5)	83.4 (76.7, 90.6)	
**LNR groups**	< 0.05	655 (76.0)	85.8 (79.8, 92.3)	**< 0.001**
	0.05 to < 0.2	128 (14.8)	90.7 (82.8, 99.4)	
	≥0.2	78 (9.0)	61.3 (45.3, 82.9)	
**LODDS**	<−1.36	569 (66.0)	84.5 (77.6, 92.1)	**< 0.001**
	−1.36 to −0.53	217 (25.2)	91.0 (85.7, 96.6)	
	> − 0.53	75 (8.7)	61.1 (44.9, 83.2)	

*ASA* American Society of Anesthesiologists, *CI* Confidence interval, *CRM* Circumferential margin, *LNR* Lymph node ratio, *LNY* Lymph node yield, *LODDS* Log odds of positive lymph nodes, *LVI* Lymphovascular invasion, *OS* Overall survival.

**Fig. 1:**
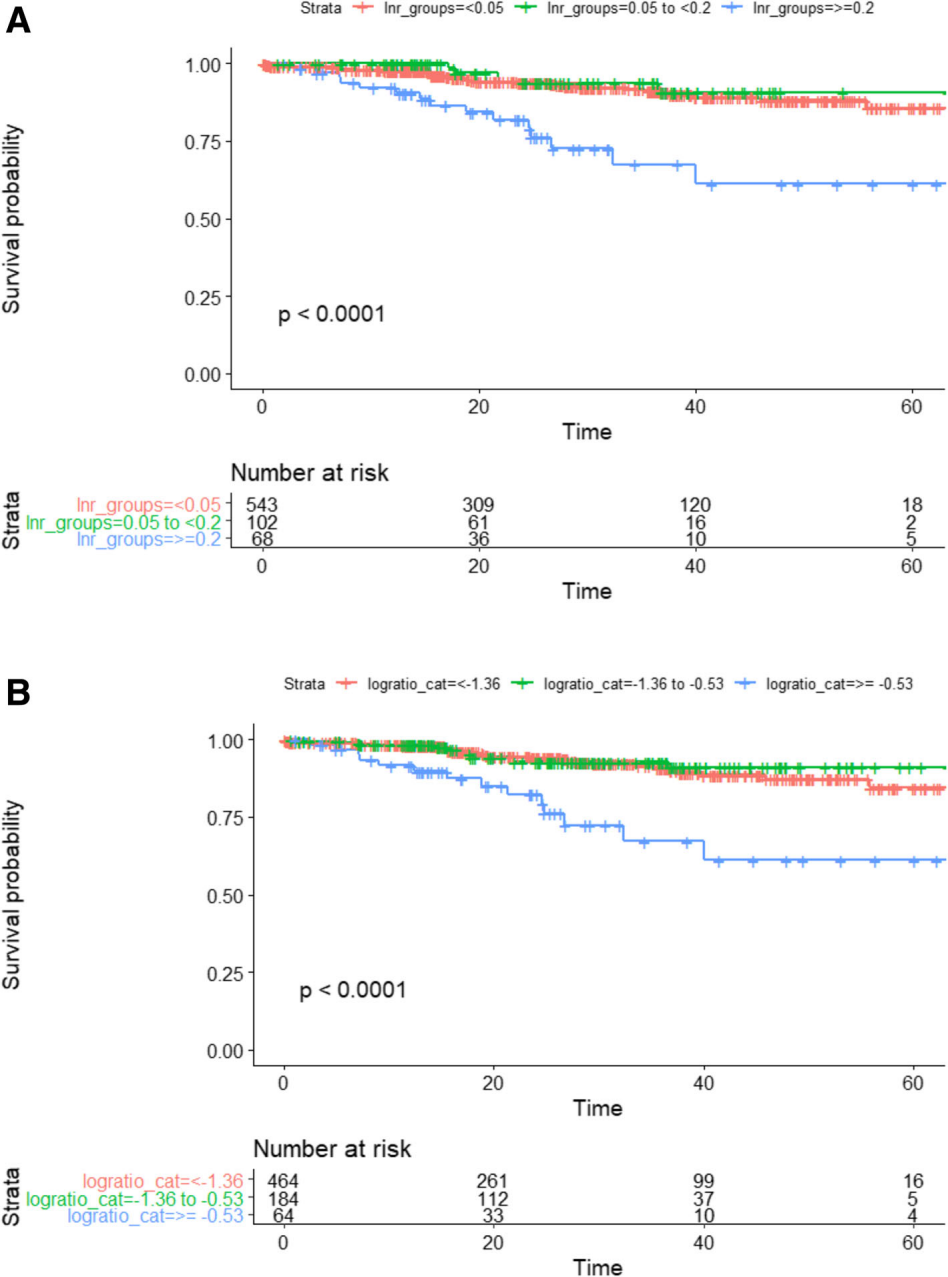
Kaplan-Meier survival curves of the patients staged by LNR and LODDS. **a.** Overall survival by LNR groups. **b** Overall survival by LODDS groups

**Fig. 2:**
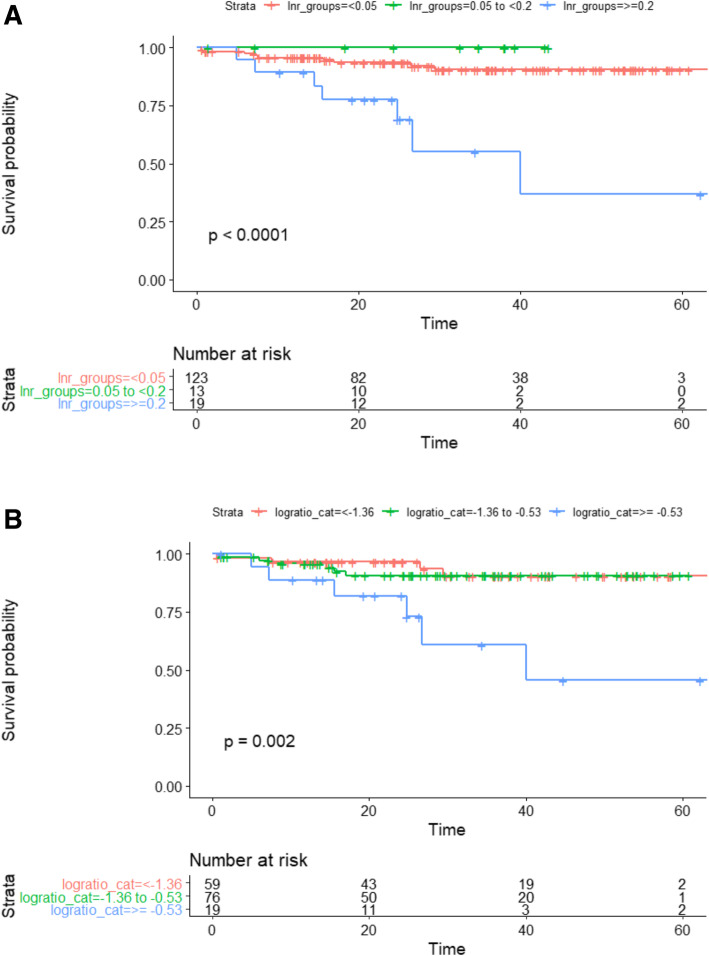
Kaplan-Meier survival curves for patients with fewer than 12 examined lymph nodes stratified by LNR and LODDS (*n* = 168). **a** Overall survival for patients with LNY < 12 by LNR groups. **b** Overall survival for patients with LN < 12 by LODDS

Five-year OS rates for women and men were 86 and 82% respectively (*p* = 0.4; Table 1). 5-year OS was reduced with increasing age, increasing T stage, N stage, ASA, and with lymphovascular invasion (LVI) (Table 1). Five-year OS rates for the different LNR groups were 85.8% for LNR_0_, 90.7% for LNR_1_, and 61.3% for LNR_2_ (*p* < 0.0001; Fig. 1). The 5-year OS stratified by nodal stages were 85.2% for pN0, 85.3% for pN1 and 71.2% pN2 (*p* = 0.01); by LODDS classification were 84.5% for LODDS_0_, 91.0% for LODDS_1_ and 61.1% for LODDS_2_ (*p* < 0.001; Fig. 1a and b). Five-year OS was not significantly different (*p* = 0.5) between patients with LNY < 12 and LNY ≥12 (85.4% vs. 83.4%) however, in the subgroup analysis of patients with LNY < 12, both LNR (*p* < 0.0001) and LODDS (*p* < 0.002) retained prognostic value for 5-year OS (Fig. 2a and b).

The univariate Cox regression analysis identified ten variables associated with survival that were statistically significant (Table 2): age ≥ 80 (*p* < 0.001), sigmoid colon tumours (*p* < 0.001), T3 stage (*p* = 0.012), T4 stage (*p* < 0.001), N2 stage (*p* = 0.002), ASA 3 (*p* = 0.003), ASA 4 (*p* < 0.001), lymphovascular invasion (LVI) (*p* = 0.017), LNR ≥0.2 (*p* < 0.001) and LODDS_2_ (*p* < 0.001). In multivariate analysis, age ≥ 80, hepatic flexure tumour site, sigmoid colon tumour site, T4 stage, and LNY ≥ 12 were identified as independent prognostic factors of OS when data was sub-grouped into LNR and LODDS categories (Table 3).

**Table 2:** Univariate analysis for overall survival

Variable		Univariate analysis	
		HR (95% CI)	***p***-value
**1st episode (*n*** **= 856)**			
**Sex**	Male	Reference group	
	Female	0.788 (0.466, 1.332)	0.37
**Age (yrs)**	< 60	Reference group	
	60–79	1.462 (0.492, 4.345)	0.494
	80+	6.129 (2.177, 17.257)	**< 0.001**
**All episodes (*n*** **= 862)**			
**Tumour site**	Caecum	Reference group	
	Ascending colon	0.961 (0.484, 1.908)	0.910
	Hepatic flexure	0.372 (0.085, 1.627)	0.189
	Transverse colon	0.757 (0.340, 1.686)	0.495
	Splenic flexure	0.450 (0.103, 1.968)	0.289
	Descending colon	0.596 (0.136, 2.607)	0.492
	Sigmoid colon	0.080 (0.018, 0.350)	**< 0.001**
	Rectosigmoid	0.534 (0.194, 1.469)	0.224
**T stage**	0–2	Reference group	
	3	2.501 (1.221, 5.123)	**0.012**
	4	10.032 (4.529, 22.221)	**< 0.001**
**N stage**	0	Reference group	
	1	0.878 (0.408, 1.891)	0.740
	2	2.852 (1.483, 5.487)	**0.002**
**ASA**	1	Reference group	
	2	3.843 (0.884, 16.715)	0.073
	3	8.702 (2.059, 36.767)	**0.003**
	4	30.668 (6.918, 135.948)	**< 0.001**
**Pathological grade**	Undifferentiated	< 0.001 (< 0.001, < 0.001)	0.996
	Poor differentiation	2.43 (1.415, 4.168)	**0.001**
	Moderate differentiation	Reference group	
	Well differentiated	0.515 (0.070, 3.776)	0.514
**LVI**	No	Reference group	
	Yes	1.916 (1.124, 3.264)	**0.017**
**CRM**	Negative > 1 mm	Reference group	
	Positive ≤1 mm	2.838 (0.382, 21.084)	0.31
	Not reported	0.764 (0.441, 1.322)	0.34
**≥ 12 LNY**	No	Reference group	
	Yes	0.803 (0.449, 1.435)	0.46
**LNR groups**	< 0.05	Reference group	
	0.05 to < 0.2	0.732 (0.287, 1.867)	0.51
	≥ 0.2	3.775 (2.063, 6.908)	**< 0.001**
**LODDS**	<−1.36	Reference group	
	−1.36 to −0.53	0.852 (0.428, 1.695)	0.65
	> 0.53	3.715 (1.974, 6.991)	**< 0.001**

*ASA* American Society of Anesthesiologists, *CI* Confidence interval, *CRM* Circumferential margin, *LNR* Lymph node ratio, *LNY* Lymph node yield, *LODDS* Log odds of positive lymph nodes, *LVI* Lymphovascular invasion.

**Table 3:** Multivariate analysis for overall survival

Variable		Multivariate analysis			
		HR (95% CI)	***p*** - value	HR (95% CI)	***p*** - value
**1st episode (*n*** **= 856)**					
**Sex**	Male		–		–
	Female		–		–
**Age (yrs)**	< 60		–		–
	60–79		–		–
	80+	6.208 (2.204, 17.484)	**< 0.001**		
**All episodes (*n*** **= 862)**		**LNR**		**LODDS**	
**Tumour site**	Caecum		–		–
	Ascending colon		**–**		**–**
	Hepatic flexure	0.212 (0.046, 0.980)	0.047	0.200 (0.043, 0.922)	**0.039**
	Transverse colon		–		–
	Splenic flexure		–		–
	Descending colon		–		–
	Sigmoid colon	0.092 (0.020, 0.421)	**0.002**	0.079 (0.017, 0.368)	**0.001**
	Rectosigmoid		–		–
**T stage**	0–2		**–**		–
	3		**–**		**–**
	4	8.92 (3.520, 22.605)	**< 0.001**	8.57 (3.394, 21.655)	**< 0.001**
**N stage**	0		-^*^		-^*^
	1		-^*^		-^*^
	2		-^*^		-^*^
**ASA**	1		-^*^		-^*^
	2		-^*^		-^*^
	3		-^*^		-^*^
	4		-^*^		-^*^
**Pathological grade**	Undifferentiated		–		–
	Poor differentiation		**–**		**–**
	Moderate differentiation		–		–
	Well differentiated		–		–
**LVI**	No		–		–
	Yes		–		–
**CRM**	Negative > 1 mm		–		–
	Positive ≤1 mm		–		–
	Not reported		–		–
**≥12 LNY**	No		–		–
	Yes	0.423 (0.220, 0.813)	**0.010**	0.382 (0.199, 0.734)	**0.004**

*ASA* American Society of Anesthesiologists, *CI* Confidence interval, *CRM* Circumferential margin, *LNR* Lymph node ratio, *LNY* Lymph node yield, *LODDS* Log odds of positive lymph nodes, *LVI* Lymphovascular invasion.

^*^ omitted due to collinearity

**Fig. 3:**
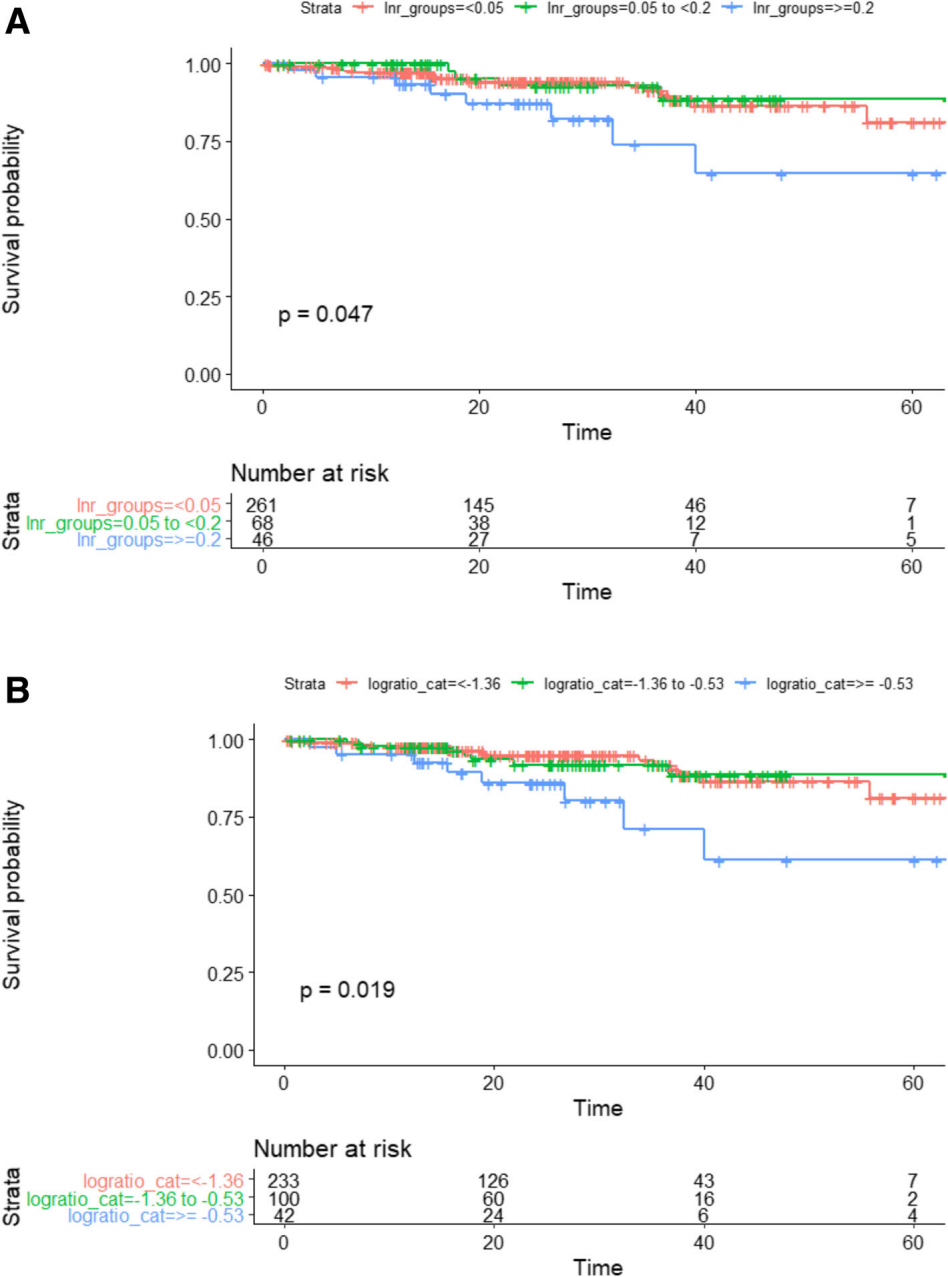
Kaplan–Meier survival curves of the patients with Stage III colon cancer stratified by LNR and LODDS. **a** Overall survival for stage III colon cancer by LNR groups. **b** Overall survival for stage III colon cancer by LODDS groups

OS rates over 5 years decreased with advancing ASA; 96% OS survival for ASA I and 46% for ASA 4 (*p* < 0.001). 619 patients (72.3%) were lymph node negative (pN0) and thus all the patients were inherently in the LNR_0_ group. Overall survival rates of node-negative patients were not significantly different between the different LODDS_0_ and LODDS_1_ groups. When OS was analysed for the 243 patients with stage III colon cancer it was observed that both LNR (*p* = 0.047) and LODDS (*p* = 0.019) were associated with decreased survival (Fig. 3a and b). During the study period 56 patients died (5.6%), 55 experienced a recurrence of their cancer (6.4%), and the mean follow-up time was 26.5 months (SD 16.2 months).

In univariate analyses of disease free survival (DFS), positive circumferential margins (*p* = 0.006), and the presence of lymphovascular invasion (*p* = 0.016) were significant, however no predictors were significant on multivariate analyses. Log rank tests of DFS survival curves showed no significance for patients staged by LNR groups (*p* = 0.13), LODDS groups (*p* = 0.77), < 12LNY (LNR groups; *p* = 0.12), < 12LNY (LODDS groups; *p* = 0.9), stage III colon cancer (LNR groups; *p* = 0.79), stage III colon cancer (LODDS groups; *p* = 0.73).

## Discussion

The current study compares the prognostic impact between LODDS and LNR in the surgical management of colon cancer. In the present study of patients with non-metastatic colonic and rectosigmoid cancers, overall survival rates were comparable between LNR and LODDS groups. Patient factors (age > 80 and ASA 3/4) and tumour factors (tumour location, tumour stage, nodal stage, LVI, LNR and LODDS) were related to 5-year OS in univariate analysis. Our finding of LNR being related to 5-year OS is similar to a Danish cohort study of 8901 patients, where LNR was superior to N-stage in differentiating overall survival in stage III colon cancer [15]. Data on LODDS in colorectal cancer remains limited and accordingly LNR is more frequently used.

A systematic review from Ceelan et al.*,* showed that LNR is a more accurate prognostic method for colorectal cancer patients and gives a superior prediction of survival to the TNM system [16, 17]. Although the LNR classification has been proven to be superior to the pN classification, there are limitations in using this for prognostic assessment. LNR has no prognostic value in node-negative cancer patients because of having the same definition of a LNR_0_ classification as pN0 classification. If there are inadequate lymph nodes harvested, then LNR is not prognostically accurate [18, 19].

LODDS is a novel indicator that improves the accuracy of lymph node evaluation for prognostic assessment irrespective of nodal positivity status and has been identified in many malignancies as a superior prognostic marker compared to LNR [10, 11]. In an analysis of 2547 curative gastric cancer patients treated with radical resection, LODDS was identified as a better prognostic indicator for overall survival than the LNR [20]. Similar findings have been mirrored for breast cancer patients [21]. In colonic cancers, LODDS was found to be an independent prognostic factor which has prognostic superiority compared to LNR or pN disease [22].

It has been proposed that the lymph node count can be used as a measure of the quality of surgery [23], however adequate lymph node harvesting cannot be achieved in approximately half of patients [24]. In the present study, 19.5% of all colon cancer resections were below the current benchmark of a minimum harvest of 12 lymph nodes; this is comparable to contemporary data from specialist centres [8, 25], but more favourable than that from other population-based studies [26, 27]. This variation in nodal harvesting can be due to patient factors (older age), operative factors (left sided/rectal operations) or the quality of the histopathological examination [28]. Studies have shown that colorectal surgeons have a higher LNY compared to those operations performed by non-specialists [29, 30]. The LNY was adequate in 80% of cases and the eleven surgeons contributing patients to our database are specialist colorectal surgeons.

Arslan et al, found that LODDS was better than LNR at providing more oncologically relevant information as it is less influenced by the LNY. Furthermore, LNR was not sufficient to stage patients when LNY was < 12 [9]. The prognostic values of both LODDS and LNR in this study were independent of the number of harvested nodes. In sub-group analysis of the patients with < 12 LNY in the present study, both LODDS and LNR were significant predictors of 5-year OS. When comparing 5-year OS between LNY < 12 to LNY ≥12, no statistical differences were found when adjusted for either LNR or LODDS.

## Conclusion

The study is the first study to examine LODDS in the Australian region (with Australia having one of the highest rates of colorectal cancer in the world) and is one of the largest published single centre series examining LODDS. This study has shown that the prognostic impact of LODDS is comparable to LNR for overall colon cancers and when stratified for stage III patients and patients with a LNY < 12. Since the prognostic information provided between the two is equivalent, LNR may be more clinically practical due to the simple calculation required. Further research is needed to assess whether the addition of the LODDS to the N category defined by the TNM would affect the selection of colon cancer patients who may most benefit from adjuvant treatments.

## Data Availability

The datasets generated during and/or analysed during the current study are not publicly available as study participants were assured raw data would remain confidential and not be shared.

## Abbreviations

AJCC: American Joint Committee on Cancer
ASA: American Society of Anesthesiologists;
CEA: Carcinoembryonic antigen
CI: Confidence interval
CRC: Colorectal cancer
CRM: Circumferential resection margin
CT: Computed tomography
DFS: Disease-free survival
LNR: Lymph node ratio
LNY: Lymph node yield
LODDS: Log odds of positive lymph nodes
LVI: Lymphovascular invasion
OS: Overall survival
TNM: Tumour nodes metastasis.
